# Genome Sequencing Reveals the Potential of *Enterobacter* sp. Strain UNJFSC003 for Hydrocarbon Bioremediation

**DOI:** 10.3390/genes16010089

**Published:** 2025-01-16

**Authors:** Gianmarco Castillo, Sergio Eduardo Contreras-Liza, Carlos I. Arbizu, Pedro Manuel Rodriguez-Grados

**Affiliations:** 1 Departamento de Agronomía, Universidad Nacional José Faustino Sánchez Carrión (UNJFSC), Lima 15136, Peru; 1735172006@unjfsc.edu.pe (G.C.); scontreras@unjfsc.edu.pe (S.E.C.-L.); 2 Facultad de Ingeniería y Ciencias Agrarias, Universidad Nacional Toribio Rodríguez de Mendoza de Amazonas (UNTRM), Amazonas 01001, Peru; 3 Programa de Genética y Mejoramiento Genético de Plantas, Universidad Nacional Toribio Rodríguez de Mendoza de Amazonas (UNTRM), Amazonas 01001, Peru; 4 Instituto de Investigación para el Desarrollo Sustentable de Ceja de Selva (INDES-CES), Universidad Nacional Toribio Rodríguez de Mendoza de Amazonas (UNTRM), Amazonas 01001, Peru

**Keywords:** NGS, genomics, microbiology, benzo[a]pyrene, hydrocarbon and bioremediation, molecular docking

## Abstract

Bioremediation induced by bacteria offers a promising alternative for the contamination of aromatic hydrocarbons due to their metabolic processes suitable for the removal of these pollutants, as many of them are carcinogenic molecules and dangerous to human health. Our research focused on isolating a bacterium from the rhizosphere of the tara tree with the ability to degrade polycyclic aromatic hydrocarbons, using draft genomic sequencing and computational analysis. *Enterobacter* sp. strain UNJFSC 003 possesses 4460 protein-coding genes, two rRNA genes, 77 tRNA genes, and a GC content of 54.38%. A taxonomic analysis of our strain revealed that it has an average nucleotide identity (ANI) of 87.8%, indicating that it is a new native Enterobacteria. Additionally, a pangenomic analysis with 15 strains demonstrated that our strain has a phylogenetic relationship with strain FDAARGOS 1428 (*Enterobacter cancerogenus*), with a total of 381 core genes and 4778 accessory genes. Orthologous methods predicted that strain UNJFSC 003 possesses genes with potential for use in hydrocarbon bioremediation. Genes were predicted in the sub-pathways for the degradation of homoprotocatechuate and phenylacetate, primarily located in the cytoplasm. Studies conducted through molecular modeling and docking revealed the affinity of the predicted proteins in the degradation of benzo[a]pyrene in the homoprotocatechuate sub-pathway, specifically hpcB, which has enzymatic activity as a dioxygenase, and hpcC, which functions as an aldehyde dehydrogenase. This study provides information on native strains from Lomas de Lachay with capabilities for the bioremediation of aromatic hydrocarbons and other compounds.

## 1. Introduction

Hydrocarbon pollution in the environment occurs through human activities or accidentally, resulting in environmental contamination [1]. Exposure to hydrocarbons can lead to a wide range of health conditions, increasing morbidity and affecting human health [2]. Hydrocarbons are classified into two groups, namely aliphatic and aromatic groups. The term “aliphatic hydrocarbon” refers to saturated compounds, such as alkanes or paraffins, which do not have double or triple bonds. “Unsaturated aliphatic hydrocarbons” have one or more double bonds (alkenes) or one or more triple bonds (alkynes). “Aromatic hydrocarbons” exhibit properties associated with the benzene ring and are known as polycyclic aromatic hydrocarbons (PAHs) [3]. Due to rapid industrialization and large-scale anthropogenic activities, the level of hydrocarbon pollution continues to increase, which is concerning for the environment. There are many techniques for hydrocarbon remediation, but the use of microorganisms has several advantages, such as cost-effectiveness, minimal or no by-products, and more [4].

Microbes are primarily used for their rapid growth and ease of manipulation [5]. The phyla Proteobacteria and Bacteroidetes, which are generally Gram-negative bacteria, have potential for hydrocarbon degradation [6]. Proteobacteria constitute one of the largest and most versatile phyla in the bacterial domain. Single bacteria or consortia of Proteobacteria have been utilized for hydrocarbon degradation, demonstrating their effectiveness in remediating these compounds [7,8]. In the last decade, several microorganisms capable of degrading PAHs have been identified and characterized. Additionally, metabolic enzymes have been isolated from microorganisms for degrading various PAH compounds, identifying diverse novel metabolic pathways, including studies on PAH catabolic operons [9].

Their genetic material has made them important for a wide variety of uses, playing a key role in natural environmental processes, mainly as regulators of the Earth’s biogeochemical cycle. They are important in applications such as the remediation of toxic and recalcitrant compounds thanks to their biodegradative nature based on their genome, and they possess different genes and pathways involved in their survival and adaptation to diverse environmental conditions, which have been useful for hydrocarbon degradation [10,11]. Studies conducted by Villela [12] analyzed the genome of bacterial consortia associated with corals, highlighting their complementary capacity in hydrocarbon degradation, with a significant degradation of PAHs and n-alkanes while also promoting additional benefits for coral health. Some microbes have the ability to remediate, detoxify, transform, and accumulate a wide range of organic and inorganic compounds [13]. On the other hand, microorganisms can be indigenous to the contaminated site or can be isolated from another location or area to be used in the contaminated site. To understand their mechanisms and, in turn, design new approaches, scientists are utilizing omics disciplines, specifically metabolomics, proteomics, transcriptomics, fluxomics, genomics, and metagenomics in bioremediation processes. By applying these sciences, it will be possible to understand their metabolic pathways, proteins, genes, and the structures of these [14]. These studies have been made possible thanks to the development of next-generation sequencing (NGS) technologies and in silico methods.

However, the most recent advances in these omics’ technologies allow for a better understanding of the physiology of microorganisms and even their metabolic functioning, leading to significant progress at the level of microbial cells and populations. These advances reveal new genes and proteins from novel microbes with useful capabilities for restoring the environment [15]. Considering the diversity of their protein composition and interactions with contaminants, new computational strategies have been implemented for studying the adsorption and remediation of environmental contaminants, such as molecular dynamics simulations and molecular docking [16]. These techniques allow us to determine the type of interaction and to understand the conformational changes in the microbial protein during its binding process [17]. Additionally, molecular simulations, such as molecular dynamics and molecular docking, have been able to overcome the limitations of experimental methodologies performed in vitro, providing a simpler and more cost-effective approach through these in silico techniques [18]. Additionally, artificial intelligence in the prediction of high-precision structures, such as AlphaFold [19], as well as the trRosetta web server [20], have provided the prediction of protein structures on a computer without any prior cost. Thanks to these computational techniques, microbial proteins can be studied in greater depth, reducing time, which is important for the prediction of enzymes in degradation pathways, as well as in domains that may serve for the degradation of hydrocarbons and other compounds and for the detection of contaminants. Additionally, these methods can be applied to environmental sciences, as well as other scientific fields [21]. Research in biological sciences is increasingly being carried out through multidisciplinary collaborations, involving professionals, scientists, engineers, and others, who analyze data from different perspectives in order to design tools that support future research [22]. Conducting research using bioinformatics tools for predicting genes and enzymes involved in microbial degradation through various pathway prediction systems has been key. Several biodegradative databases, including EAWAG-BBD (Biocatalysis and Biodegradation Database), the microbial plastic biodegradation database, ONDB (Organonitrogen Degradation Database), OxDBase (a database of biodegradative oxygenases), Aromadeg (a database of aromatic hydrocarbons), and RHObase (a database of ring-hydroxylating oxygenases), have been developed for bioremediation and biodegradation studies [23]. Here, we use a modern database with the capability to predict genes and enzymes involved in hydrocarbon degradation, featuring experimentally validated data on enzymes through orthology.

In this study, de novo genome sequencing was performed on an enterobacterium isolated from soil samples of the rhizosphere of tara plants (*Caesalpinia espinosa*) from the Lomas de Lachay National Reserve. Genes involved in the degradation of aromatic hydrocarbons were identified. In addition, a proteome prediction was conducted followed by the construction of three-dimensional molecular structures of putative proteins involved in aromatic hydrocarbon bioremediation. This involved deep learning modeling for molecular docking with the benzopyrene molecule to assess their potential interaction against this compound. To the best of our knowledge, this work provides the first report on bacteria isolated from the Lomas de Lachay reserve and on the genomics and molecular biology of *Enterobacter* sp. UNJFSC003 in response to polycyclic hydrocarbons.

## 2. Materials and Methods

### 2.1. Isolation of Strain, Extraction of DNAg, and Genome Sequencing

The sample was collected in Lomas de Lachay (11°22′39.2″ S, 77°21′34.6″ W), Province of Huaura, Lima, Peru. One kilogram of soil was collected in sterile bags at a depth of 30 cm and 4 cm from the root of the tara plant (*C. espinosa*). To isolate the bacteria, 1 g of soil was mixed with 9 mL of sterile 0.5% sodium chloride (NaCl), serial dilutions were prepared, and these dilutions were plated on Tryptic Soy Agar (TSA) medium. The plates were incubated at 30 °C for 48 h. To purify the bacteria, subculturing was performed on TSA medium. Colony morphology on the plate and Gram staining were identified. Additionally, biochemical tests were conducted to establish its identity.

The pure sample was selected for DNA extraction using the commercial EZ-10 Spin Column Bacterial Genomic DNA Miniprep Kit (Bio Basic, Ontario, Canada). Genome sequencing was performed using the Illumina NovaSeq system at a foreign company (Macrogen, Republic of Korea). The read quality was assessed using FastQC v0.12.1 [24]. Additionally, the reads were trimmed and filtered (Q-25) using trimmomatic v0.36 [25] and fastp v0.20.1 [26] with default parameters.

### 2.2. Genome Assembly, Annotation, and Functional Analysis

**Figure 1:**
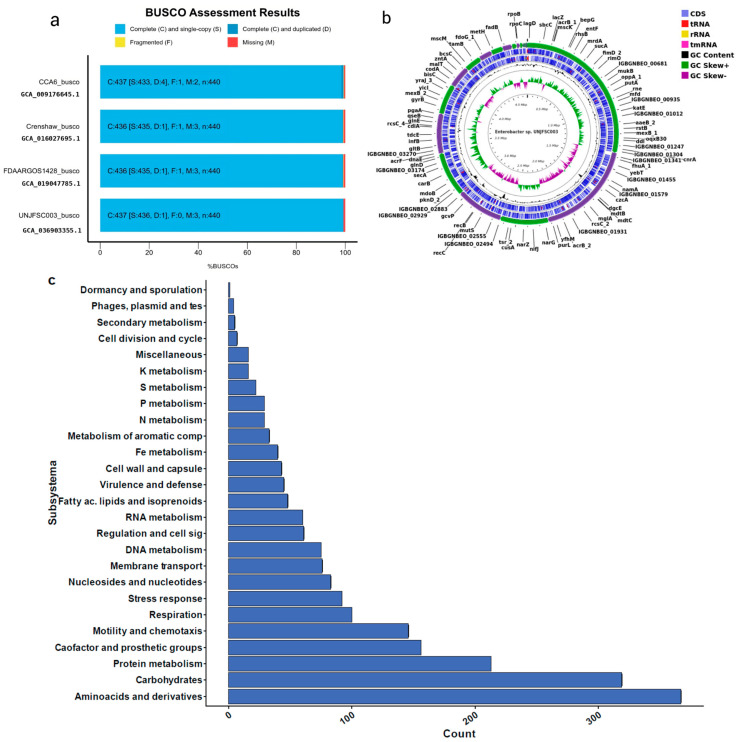
Genomic results of *Enterobacter* sp. (**a**) Comparison of BUSCO analysis between UNJFSC003 and the strains CCA6, Crenshaw, and FDAARGOS1428. (**b**) Genomic map of *Enterobacter* sp. UNJFSC003. (**c**) Subsystem distribution in UNJFSC003.

To obtain the genome of *Enterobacter* sp. strain UNJFSC 003, a de novo assembly was performed using the Unicycler v0.5.0 assembly algorithm [27]. This assembly algorithm was selected due to its distinct methodological approaches, which allow for the optimization of the SPAdes assembly, thus obtaining longer contigs used for this genome. After this, we used QUAST v5.2.0 [28] to evaluate the assembly statistics. Subsequently, the assembly was subjected to the BUSCO v5.2.2 strategy [29] to assess the completeness of our genome assembly compared to three other strains deposited in the NCBI Datasets Genome (Figure 1a) using previous scripts (https://github.com/GianmarcoCastillo/Genome-UNJFSC003/tree/main, accessed on 31 October 2024). Additionally, MiGA v1.3.9.0 was used to evaluate the quality, contamination, and identification of the genome [30].

Genome annotation was performed using Prokka v1.14.5 [31] and RAST [32], and a genomic map was created using the Proksee tool [33]. Moreover, a pie chart of the genome subsystem was created from the annotation results using matplotlib v3.8.4 [34] and pandas v1.5.3 packages in Python v3.10.12. The predicted genes were functionally characterized using the COG (Cluster of Orthologous Genes) tool, orthology assignment through the eggNOG-Mapper tool [35], and pathway reconstruction was performed using the KEGG (Kyoto Encyclopedia of Genes and Genomes) [36] and BlastKOALA [37] databases. Furthermore, the presence of resistance genes for antibiotics was determined using the abricate v1.0.0 approach available at (https://github.com/tseemann/abricate, accessed on 31 October 2024) using the contigs of our strain.

### 2.3. Comparative Genome Analysis, Molecular and Pangenome Confirmation

In the genome comparison and molecular confirmation of *Enterobacter* sp. UNJFSC003, the whole-genome sequence (WGS) of the isolate was used. An average nucleotide identity (ANI) analysis was performed between *Enterobacter* sp. UNJFSC003 and 15 other Enterobacter strains obtained from the NCBI genome database. This analysis was calculated using the fastANI v1.34 tool [38]. Subsequently, a heatmap of clusters of the 16 genomes was generated using the ANIclustermap v1.3.0 tool [39] to assess the diversity of taxonomic and phylogenetic lineages among the genomes. A pangenome analysis of 16 different Enterobacter strains was conducted using Roary v3.13.0 [40], utilizing GFF3 files obtained from Prokka. For this analysis, the following parameters were used: a BLASTP identity threshold of 90%, clustering in groups of 100,000 sequences, and separation of paralogs. The results were visualized in a phylogenomic tree, heat map, and a histogram using the python script from this github repository (https://github.com/sanger-pathogens/Roary, accessed on 31 October 2024).

### 2.4. Prediction of Hydrocarbon-Degrading Genes and Enzymes

In the prediction of genes and enzymes involved in hydrocarbon degradation, the A Curate Hydrocarbon Aerobic Degradation Enzymes and Genes Database (HADEG) [41] was used. This database contains experimentally validated protein and gene sequences involved in the aerobic degradation of hydrocarbons. The analysis used the .faa files obtained from Prokka and the tool proteinortho v6.0.33 [42] to detect orthologous proteins capable of degrading hydrocarbons within the genome of *Enterobacter* sp. UNJFSC003. In addition, the HADEG.R script present on github (https://github.com/jarojasva/HADEG, accessed on 31 October 2024) and the ggplot2 [43] in R v4.4.0 package was used, resulting in a heat map, a bubble diagram, and a bar graph of the genes and proteins involved in the degradation of hydrocarbons.

### 2.5. In Silico Protein–Protein Interaction and Subcellular Localization of Hydrocarbon-Degrading Proteins

The protein interaction network was evaluated by subjecting the proteins involved in aromatic hydrocarbon degradation to the STRING database with a high confidence score threshold (>0.7) [44]. The database includes both direct (physical) and indirect (functional) associations through computational predictions. The interaction results of all proteins were visualized using Cytoscape v3.10.2 [45]. For the protein localization within the cell of the *Enterobacter* sp. UNJFSC003 genome, the bioinformatics tool PSORT v3.0.3 [46] was employed. The results were visualized in a heatmap using the Pheatmap package [47] in R v4.4.0 [48].

### 2.6. Molecular Modeling, Model Validation, and Molecular Docking

The proteins presumed to be involved in aromatic hydrocarbon degradation, specifically the hpc gene cluster predicted by the HADEG database, were selected. A functional analysis of these hpc proteins was conducted by classifying them into families and predicting domains and active sites in each protein using the bioinformatics tool InterProScan [49]. For the 3D molecular structure modeling, proteins were modeled using the trRosetta server [20]. The models were validated using PDBsum [50], ERRAT [51], and ProSA Web [52]. Additionally, a signal peptide analysis was performed using SignalP v.6 [53] for each predicted protein, and physicochemical analyses were conducted using the Expasy ProtParam tool [54]. The molecular docking analysis involved blind docking using the AutoDock Vina v1.1.2 software [55], facilitated by the graphical interface AutodockTools v1.5.6 [56]. This approach was used to study protein interactions and conduct preliminary analysis with the ligand benzopyrene (https://pubchem.ncbi.nlm.nih.gov/compound/2336, accessed on 31 October 2024) sourced from PubChem. Beforehand, hydrogens were added to the protein, charges were assigned using the Kollman method, and non-polar hydrogens were subsequently removed. The grid coordinates used for all proteins were as follows: x = 16.305, y = 20.737, and z = 1.165, with dimensions of 126 in each direction (x, y, z) and a spacing of 0.631 Å. Additionally, for the ligand molecules, energy minimization was performed beforehand using Avogadro v1.2.0 [57]. Hydrogens were added, Gasteiger charges were assigned, and non-polar hydrogens were subsequently removed. The results of the molecular docking were visualized using PyMOL v3.0.2 [58].

## 3. Results

### 3.1. Isolation of the Strain and Genome Characteristics of Enterobacter sp. UNJFSC 003

The pure strain was successfully isolated on TSA culture medium. Additionally, the colony exhibited a transparent color and a star-shaped form. The Gram stain revealed that it was a Gram-negative bacterium. The biochemical profile included a growth assay [59] on MacConkey and eosin methylene blue (EMB) media, the fermentation of glucose (+), gas production (+), oxidase negative (−), catalase positive (+), and motility test (+).

The total number of raw reads was 10,914,547 sequences, with an average length of 151 base pairs (bps), a GC content of 54%, and a total sequencing output of 1.6 gigabytes (GB). The results of the cut made can be seen in Table 1.

The de novo assembly was conducted with the unicycler program because it can assemble Illumina read assemblies by working as a SPAdes optimizer, obtaining better results from N50 and longer contigs. We tested different k-mers (from 27 to 127). In the assembly quality assessment (Quast), we obtained statistical results of 51 contigs (≥ bps), with a GC content of 54.38%. The longest contig obtained was 4,663,955 base pairs (≥50,000 bps). Additionally, the N50 result was 415,254 and the L50 was three. In the taxonomic classification analysis of the *Enterobacter* sp. UNJFSC003 genome assembly conducted by MiGA, the results indicate that our strain belongs to the family Enterobacteriaceae, with a *p*-value of 0.0025. It is likely to belong to the genus Enterobacter (*p*-value: 0.02) and possibly to the species *E. asburiae* NZ CP065693, with an average nucleotide identity (ANI) of 87.85%. Additionally, MiGA provided other results such as the consistency and quality of essential genes, with a genome quality value of 93.6%, completeness of 98.1%, and contamination of 0.9% (Table 2) Finally, in the genome annotation, the following results were obtained: a total genome size of 4,798,267 base pairs (bps), 4460 protein-coding sequences, two rRNAs, 77 tRNAs, and one tmRNA. Additionally, a functional analysis of genes and orthologous proteins yielded a total of 2671 orthologous proteins and 1487 KEGG-annotated metabolic proteins obtained through prior analysis using KOALA-KEGG. Other results include 4349 orthologous gene clusters, 4186 Pfam annotations, 2540 gene ontology assignments, 77 carbohydrate-active enzymes, and the reconstruction of metabolic networks (BiGG), with a total of 1064 nodes. Furthermore, our strain of *Enterobacter* sp., UNJFSC 003, in the prediction of antibiotic resistance, a total of four antibiotic resistance genes could be found with an identity greater than 80%. The resistance genes predicted by Abricate, using the corresponding database [60], were two in fenicol, one in fosfomycin and one in cephalosporin. These results were summarized using the EggNOG-Mapper online tool and are presented in Table 2.

The genome sequencing data are available in GenBank at NCBI under the accession number JBAKHK000000000.

### 3.2. Comparative Analysis of the Complete Genome of Enterobacter sp. UNJFSC 003

**Figure 2:**
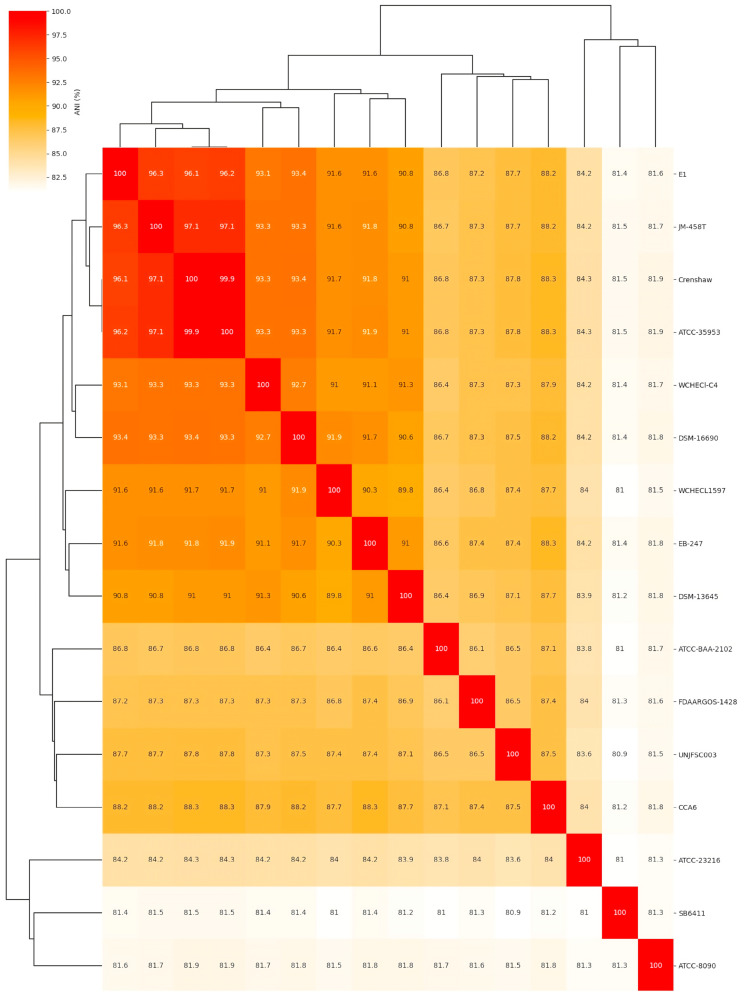
The heatmap of average nucleotide identity (ANI) values of the isolated genome of *Enterobacter* sp. UNJFSC003 against 15 reference genomes. The numbers represent ANI (%) values between genome sequences. The NCBI GenBank reference strains used in this study are listed in Table 3.

The comparative analysis of the complete genome based on the average nucleotide identity (ANI) of 16 different Enterobacter strains showed that *Enterobacter* sp. UNJFSC003 was closely related to nine Enterobacter strains with ANI values ranging from 87.8 to 87.3 as the upper limit, and the other five strains had ANI values ranging from below 86.5 to 80.9 (Figure 2 and Table 3). Our strain UNJFSC003 and the Crenshaw strain of *E. asburiae* with GenBank accession GCA_016027695 had a maximum ANI value of 87.8%. This ANI value indicates a relationship between our strain and this bacterium with an ANI value below 95%, considering that the threshold for classifying strains is (≥) 95% ANI [61]. Based on this, we can suggest that our strain is a new native Enterobacter isolated from soil samples from Lomas de Lachay [62,63].

### 3.3. Analysis of the Pangenome of Enterobacter sp. UNJFSC 003

**Figure 3:**
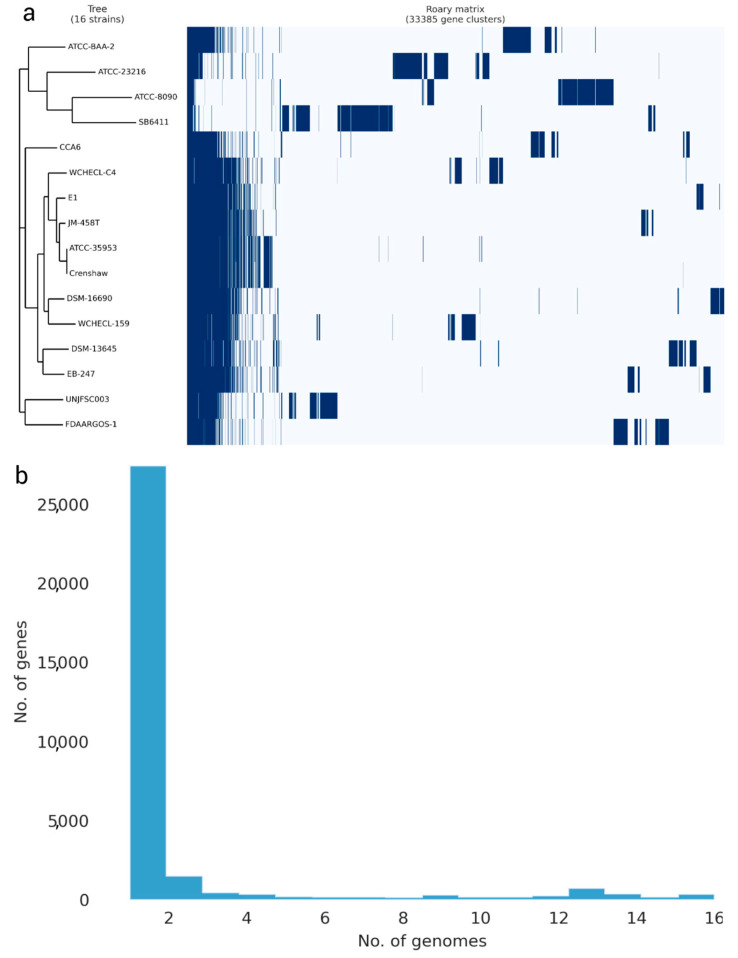
The pangenome of 16 *Enterobacter* strains was determined using the Roary matrix. A total of 33,385 orthologous protein-coding genes were found. (**a**) Heatmap showing the presence (dark blue) or absence (light blue) of genes in each of the 16 strains. A phylogeny constructed based on core genes (left) and genome strain names (right). (**b**) Histogram showing the distribution of genomes per gene.

The pangenome model developed with the participation of 16 Enterobacter strains, including 12 Enterobacter strains, three strains of other Enterobacteriaceae species, and our strain, indicated a close genetic relationship between UNJFSC003 and FDAARGOS-1 (*E. cancerogenus*). Both strains grouped into a single node, indicating their similarity in identical gene clusters. Ten other Enterobacter strains with less variation in genes were observed in the matrix and separated into another node. The three Enterobacteriaceae strains, including *L. adecarboxylata* strain ATCC2316, *K. spallanzanii* strain SB6411, and *C. freundii* ATCC8090, exhibited greater variation, along with *Enterobacter soli* strain ATCC-BAA-2. The pangenome of the 16 Enterobacter strains consists of 381 core genes, 75 soft core genes, 4778 shell genes, and 38,044 cloud genes, totaling 43,278 genes. Interestingly, *Enterobacter* sp. UNJFSC003 harbors unique gene clusters presumably present with *E. cancerogenus* strain code FDAARGOS-1 (Figure 3).

### 3.4. The Strain UNJFSC 003 Harbors Genes Encoding Enzymes for the Bioremediation of Hydrocarbons

**Figure 4:**
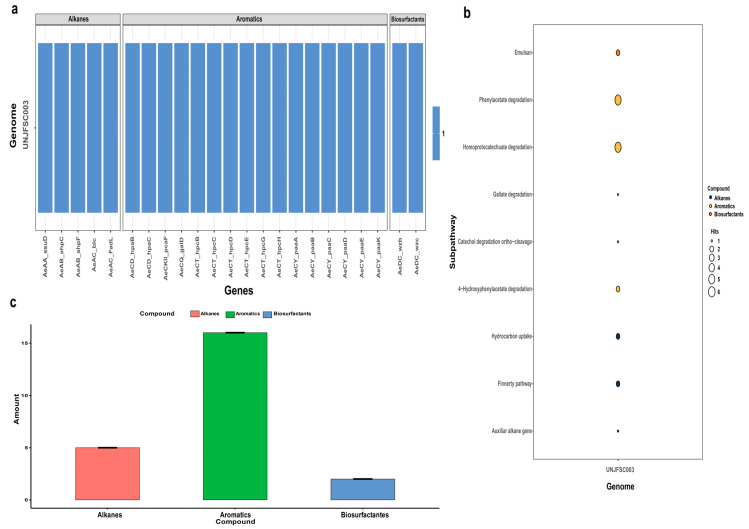
Prediction of enzymes in hydrocarbon degradation by the HADEG database. (**a**) Heatmap of all 21 enzymes of UNJFSC003 involved in the degradation of hydrocarbons, including alkanes, aromatics, and biosurfactants. (**b**) Bubble chart showing the distribution of sub-pathways of the HADEG-validated enzymes. (**c**) Histogram displaying the number of enzymes for each type of hydrocarbon and biosurfactant.

The HADEG database identified our strain as a potential organism for developing hydrocarbon bioremediation methods, finding two types of hydrocarbons, namely alkanes (a total of five) and aromatics (a total of 16). Additionally, two enzymes for biosurfactant production, all determined through an orthology analysis, were identified (Figure 4). The orthology analysis performed by proteinortho with the HADEG database identified proteins involved in the metabolic sub-pathways of the genome. This detailed and specific analysis identified two different types of hydrocarbons (alkanes and aromatics) and biosurfactants, as well as the specific number of enzymes for the bioremediation of aromatic hydrocarbons and alkanes (Figure 4).

### 3.5. In Silico Protein–Protein Interaction and Heat Map of Proteins Involved in Bioremediation

**Figure 5:**
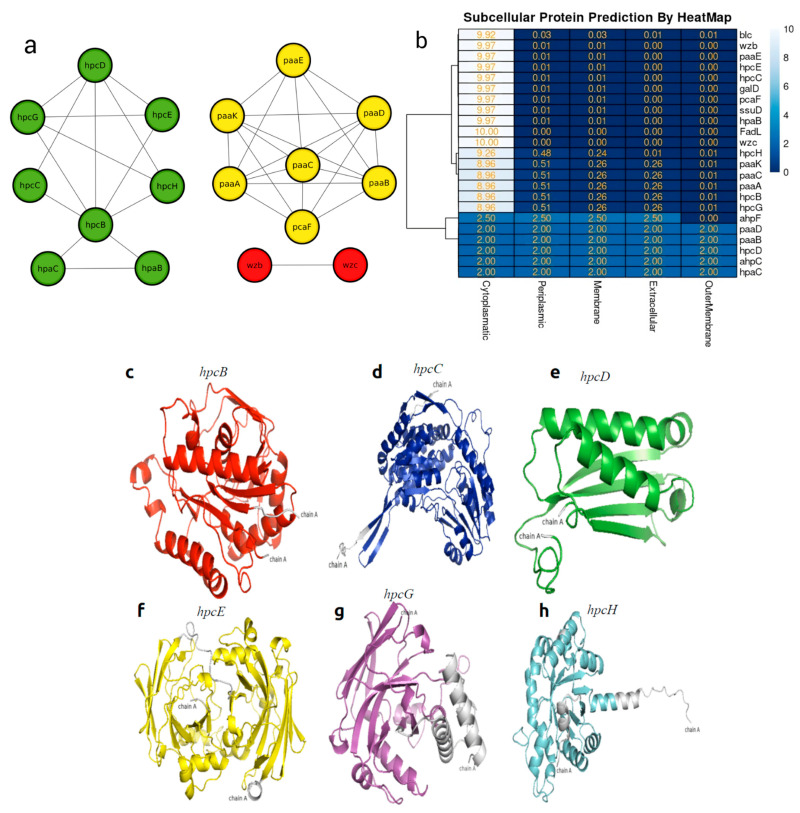
(**a**) Heat map showing the subcellular localization of the 21 enzymes. (**b**) Predicted network interactions of proteins involved in hydrocarbon degradation, with protein clusters marked in green (hpc and hpa), yellow (paa and pca), and red representing the two biosurfactant proteins. (**c**) Model of hpcB. (**d**) Model of hpcC. (**e**) Model of hpcD. (**f**) Model of hpcE. (**g**) Model of hpcG. (**h**) Model of hpcH.

The analysis of protein–protein interactions indicated possible interactions among the various proteins involved in hydrocarbon bioremediation. Indeed, the interaction among enzymes involved in the bioremediation of aromatic hydrocarbons ensures their mutual connection for each different HC, such as hpc genes totaling sic in the homoprotocatechuate pathways, paa genes totaling six in the phenylacetate pathway, and hpa genes in aerobic alkane degradation (Figure 5a). In the localization of the proteins involved in hydrocarbon bioremediation, it can be observed that 17 proteins predicted by PSORT are located in the cytoplasm and six are located in different sites of the bacterial cell (Figure 5b).

### 3.6. Molecular Modeling and Docking Analysis

The three-dimensional molecular structures of six proteins were constructed using deep modeling and Rosetta. These proteins are related to the bioremediation of aromatic hydrocarbons, specifically the proteins predicted for the bioremediation of aromatic hydrocarbons involved in the sub-pathways of homoprotocatechuate. According to the validation, all the models can be considered reliable, as the Z-score, Ramachandran analysis, and ERRAT provided satisfactory or good scores. In this context, the best model corresponds to hpcB, based on the following results obtained: 95.4% in the Ramachandran plot, a Z-score of −11.36, and an ERRAT score of 97.87. On the other hand, hpcD and hpcE correspond to the worst models, with Ramachandran plot results of 94.6%, a Z-score of 4.79, and an ERRAT score of 83.46.

On the other hand, none of the proteins have shown a signal peptide, indicating that they are all intracellular, with each protein containing between 126 and 488 amino acids. Additionally, all the obtained models displayed a monomeric folded structure. In the six predicted proteins, all possess active sites for the degradation of polycyclic aromatic hydrocarbons (Table 4).

The hpcB model has a topology of 16 α helices, 23 β sheets, and 33 coils. In the respective molecular docking analysis, this predicted protein obtained an energy value of −10.1 kcal/mol, interacting with the amino acids TRP58, ASN67, LEU121, LEU123, and GLU124. Conversely, the hpcC model has a topology of 14 α helices, 19 β sheets, and 34 coils. In the respective molecular docking analysis, this predicted protein obtained a value of −10.7 kcal/mol, interacting with the amino acids GLN95, LEU267, PHE268, SER272, and GLN316 and does not form any hydrogen bonds. Additionally, hpcD has a topology of three α helices, six β sheets, and 10 coils. In the respective molecular docking analysis, this predicted protein obtained an energy value of −9.7 kcal/mol, interacting with the amino acids GLY81, GLU82, PHE85, and PHE105; in the other analyses, hpcE has a topology of 18 α helices, 12 β sheets, and 32 coils. In its molecular docking analysis, this protein obtained a value of −12.0 kcal/mol, interacting with the amino acids TYR28, ASN29, THR30, TYR103, ARG315, and TYR314; additionally, hpcG has a topology of 11 α helices, 10 β sheets, and 19 coils. In the respective molecular docking analysis, this predicted protein obtained an energy value of −9.1 kcal/mol, interacting with the amino acids TYR128, ASP181, LEU185, HIS210, THR161, and despite having a histidine amino acid, it does not form any hydrogen bonds. Finally, in the group of proteins that degrade homoprotocatechuate, hpcH has a topology of 12 α helices, eight β sheets, and 21 coils. In the respective molecular docking analysis, this predicted protein obtained an energy value of −9.0 kcal/mol, interacting with the amino acids TRP19, GLY21, LEU212, and VAL234. Similar to hpcG, it also does not form any hydrogen bonds despite the presence of histidine.

## 4. Discussion

The microorganisms with an efficient biodegradation of aromatic hydrocarbons belonging to *Enterobacter* sp. and *E. xianfagensis* [64,65] have been reported for their ability to degrade both aromatic hydrocarbons and petroleum. This includes real-time experimental methods and computational molecular docking. Additionally, studies have explored their production of biosurfactants like rhamnolipids, as seen in *E. asburiae* [66]. In another study, *E. ludwigii* strains found as endophytes in plants were discovered to be capable of degrading hydrocarbons from the rhizosphere [67]. In this study, the strain of Enterobacter belonging to the species *E. ludwigii* possesses specific genes for the degradation of both alkanes and aromatic hydrocarbons, as well as genes involved in biosurfactant production. Thus, *Enterobacter* sp. UNJFSC003 exhibited a variety of enzymatic activities, including dioxygenase activity attributed to the LigB domain in hpcB. This family of enzymes performs the cleavage of aromatic rings as a key function in the degradation of these compounds. They also participate in the metabolism of halogenated aromatic compounds [68]; in addition, aldehyde dehydrogenase in hpcC functions enzymatically by oxidizing a broad spectrum of aliphatic and aromatic aldehydes, utilizing NAD+ and NADP+ as cofactors. These enzymes play pivotal roles in different stages of hydrocarbon degradation [69] and also play roles in the degradation of compounds such as pesticides and other toxic substances [70]. The enzymatic function of CHMI (5-carboxymethyl-2-hydroxymuconate isomerase) in the decomposition of aromatic compounds is studied within the context of hpcD. The structure of this protein was predicted using artificial intelligence through AlphaFold 2, developed by DeepMind [71], where users are studying the bioremediation of aromatic compounds in Pseudomonas aeruginosa PAO1. Additionally, they conducted an experimental study cloning the gene in *Escherichia coli* to produce large quantities of this protein and determine its properties [72]. Finally, the hydrolase activity in hpcE involves two present domains of hydrolase, whereas hpcG has a single present domain of hydrolase. The latter catalyzes the hydration of a carbon–carbon double bond without the assistance of any cofactor. The crystal structure of hpcG features the FAH fold, which is quite common among enzymes involved in the degradation of aromatic compounds [73], Lastly, hpcH also obtained a functional domain in the degradation of aromatic compounds according to the analyses conducted by InterProScan [49]. These studies were conducted using the proteome obtained from annotation developed by Prokka, orthology methods employing the Proteinortho tool, the HADEG database, and active sites identified by InterProScan.

In the present study, our bacterium isolated from soil samples from Lomas de Lachay was observed to form star-shaped colonies and was classified as Gram-negative by Gram staining. Its genome was sequenced using the Illumina NovaSeq system (Macrogen, Republic of Korea). The subsequent taxonomic identity analysis characterized this bacterium as belonging to the genus *E. asburiae* with an ANI value of 87.85%. However, the taxonomic classification of a microbial strain in the genomic era generally requires sharing an average nucleotide identity (ANI) value of >95% across multiple aligned genes [74]. This suggests that our isolated strain does not belong to the genus *E. asburiae* but may instead represent a new Enterobacter species. It maintains a close nucleotide content relationship with *E. asburiae* and possesses unique gene clusters similar to those found in *E. cancerogenus*, as observed in the pangenome analysis results with 16 Enterobacter strains. In another study, the genome of strain WCHECI-C4 has an ANI of only 93.34%, leading to its classification as a new species within the genus Enterobacter, named *E. chengduensis* sp. nov. [75]. The draft genome of strain UNJFSC003 has a size of 4,798,267 base pairs, 4460 protein-coding genes, and a GC content of 54.38%. This GC content aligns with findings from the work of [76]. *Enterobacter* sp. UNJFSC003 showed a genome completeness of 98.1%, contamination of 0.9%, and quality of 93.6%. In another study, *Enterobacter* sp. strain RIT-637 with antibacterial activities showed a genome completeness of 99.96% and contamination of 2.08% [77]. A total of 2671 genes associated with KEGG pathways were obtained. This represents a significantly larger number of genes compared to the study by Indugo [78], who found a total of 1567 genes in KEGG pathways. The classification included 2540 orthologous genes, 77 active enzymes in carbohydrates, 1064 metabolic networks in total, and 4329 clusters of orthologous genes. Interestingly, studies by Szczerba [79] found a new strain of the genus Enterobacter isolated from a cow rumen, *E. aerogenes* LU2, with protein-coding genes involved in carbohydrate metabolism pathways, amino acid transport, carbon source transport, and nitrogen source transport. Furthermore, the genome of *Enterobacter* sp. UNJFSC003 showed capabilities for hydrocarbon bioremediation and the production of low-molecular-weight (LMW) biosurfactants similar to emulsan. Microbial biosurfactants are produced as secondary metabolites or through enzymatic processes, well-known for their ability to reduce surface tension [80]. Due to the analyses conducted, we found biotechnological potential in this genus of bacteria. In the study by Peng [81], they isolated *Enterobacter* sp. T2 from contaminated sludge capable of rapidly degrading tetrabromobisphenol (TBBPA) via a key protein, haloacid dehalogenase, responsible for TBBPA degradation. In another study by El-belgati [82], they observed highly significant positive correlations between genetic expressions and increased concentrations of heavy metals, indicating that gene expression was induced by higher concentrations of heavy metals. These facts indicate the great potential that Enterobacter strains have for degrading hydrocarbons, heavy metals, and other compounds, thanks to studies using next-generation sequencing technologies.

Through the analysis of hydrocarbon biodegradation, the results suggested the efficiency of our strain UNJFSC003, identifying enzymes related to hydrocarbon bioremediation, including alkanes and aromatics. Such a result is associated with the hydrocarbon degradation effect employed by three strains of Pseudomonadota bacteria isolated from a century-old abandoned oil exploration well, analyzing their proteome and using the same gene and enzyme database for aerobic hydrocarbon degradation, finding similar types of hydrocarbons present in our study, among other types of hydrocarbons [83]. Here, the in silico analysis of the putative protein interaction networks involved in hydrocarbon bioremediation, using the STRING database, revealed potential links between the homoprotocatechuate degradation pathway, specifically hpcB, and the hydroxyphenylacetate degradation system, hpaB and hpaC. An additional connection was found between the catechol degradation pathway, specifically pcaF, and all predicted proteins in the phenylacetate degradation pathway. Additionally, the two biosurfactant-producing proteins interacted with each other without revealing links to other proteins, maintaining their phylogenetic coexistence. Interactions of proteins involved in bioremediation have already been reported in other bacteria isolated from wastewater, such as *Bacillus cereus* AA-18 [84]. Additionally, another study on cadmium bioremediation by Khan [85] shows a similar interaction network to the hpc cluster in our study, involving binding proteins such as YfiY, yfmF, and yfmE. In the subcellular prediction made by the online tool PSORTb v3.0, 17 proteins were located in the cytoplasm, while the rest were found in various cellular regions. There are many methods for predicting the subcellular localization of microbial proteins, ranging from genome-based predictions using PSORTB to metagenome-based predictions with PSORTm. These methods also include the identification of new markers in aquatic and terrestrial microorganisms [86,87].

**Figure 6:**
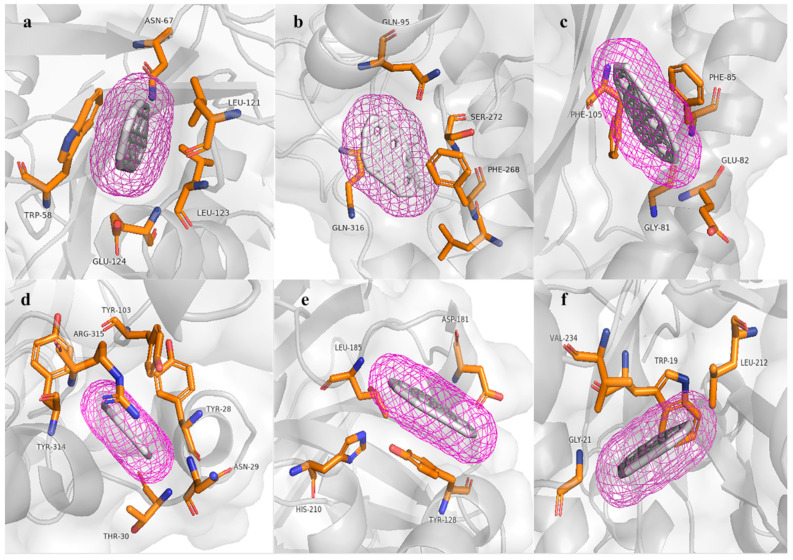
Results of molecular docking with the HAP (benzo[a]pyrene) molecule. (**a**) Active site amino acids of hpcB. (**b**) Active site amino acids of hpcC. (**c**) Active site amino acids of hpcD. (**d**) Active site amino acids of hpcE. (**e**) Active site amino acids of hpcG. (**f**) Active site amino acids of hpcH.

The molecular models of key enzymes (Table 4 and Figure 6) involved in the bioremediation of aromatic hydrocarbons showed highly conserved amino acids in the active sites. These results, along with the Ramachandran plot percentage, support the reliability of the modeled structures. Furthermore, all models did not form hydrogen bonds but showed high affinity energy with the benzopyrene molecule at the active sites of all modeled proteins through deep modeling, with affinity energy values ranging from −9.0 kcal/mol to −12.0 kcal/mol. This provides information about the structure and function of the hpc enzyme cluster. Therefore, the 3D models of hpc cluster enzymes strongly support their function in the bioremediation of aromatic hydrocarbons, as annotated from the genes of *Enterobacter* sp. UNJFSC003.

Combining genomic analysis and molecular modeling, this research has contributed to a better understanding of the great potential of strain UNJFSC 003 isolated from soil samples from Lomas de Lachay. Genomic analysis provides information about the genes involved in the bioremediation of aromatic hydrocarbons, alkanes, and biosurfactant production. Lou [88] revealed information about the genome of various genetic functionalities, specifically the metabolism of phenanthrene and pyrene degradation in two new bacterial strains, *Klebsiella michiganensis* EF4 and *K. oxytoca* ETN19, isolated from a soil sample contaminated with PAHs. In the molecular docking analysis, benzopyrene was used as the ligand. Benzopyrene is listed among the priority pollutant polycyclic aromatic hydrocarbons by the United States Environmental Protection Agency [89]. Benzopyrene has been detected in industrial waste, diesel exhaust gasses, charcoal-based foods, and elevated levels of cigarette smoke, and it is also known as a recalcitrant molecule [90]. Gram-negative bacteria with the ability to degrade polycyclic aromatic compounds have already been reported, such as in the study by Wang and Tam [91], where the bacterium Cycloclasticus was confirmed to be a ubiquitous degrader of marine PAHs, even in subterranean marine environments. In fact, a limited number of previous studies have reported PAH degradation capabilities in the phylum Proteobacteria, including pathogenic bacteria.

## 5. Conclusions

We evaluated the bioremediation capability of aromatic hydrocarbons by *Enterobacter* sp. UNJFSC003 through computational methods. This strain was isolated from a soil sample from the rhizosphere of the tara plant (*C. espinosa*) in Lomas de Lachay. The findings revealed genes with the ability to degrade aromatic hydrocarbons, alkanes, and produce biosurfactants. The analysis of the draft genome revealed these genes, as well as a new species of Enterobacter, due to the results showing an average nucleotide identity (ANI) of less than 95%. In the pangenome analysis, it was revealed that our strain UNJFSC003 harbors unique gene clusters similar to those found in strain FDAARGOS-1, with a total of 381 core genes and 4778 shell genes. The 3D structural analysis of modeled proteins from the hpc gene cluster related to the bioremediation of aromatic hydrocarbons has shown highly conserved regions, particularly in their active sites, strongly supporting their predicted functions in the degradation of polycyclic aromatic compounds. The computational prediction of all modeled monomers by deep learning using trRosetta is also noteworthy, specifically hpcB and hpcC. This study provides information to obtain, in the future, a better experimental understanding of native strains from Lomas de Lachay and their capability in the bioremediation of aromatic hydrocarbons and other compounds.

## Figures and Tables

**Table 1:** Trimming Illumina reads.

Quality Control FastQ	Results
High-quality readings	10,613,744 (97.24%)
Readings due to low quality	19.324 (0.18%)
Contained too much pollution	336 reads (0.001539%)
Readings too short	5.8520 (0.026808%)
Cut-out adapters	Yes

**Table 2:** Genome properties and characteristics of the draft genome of *Enterobacter* sp. UNJFSC 003.

Properties and Characteristics	Total
Sequence size genome	4,798,267
No. of scaffolds	
Contigs >= 0 bps/1000 bps/50,000 bps	51/28/12
N50/L50	415,254/3
No. of CDS	4460
No. of rRNA/tRNA/tmRNA	2/77/1
GC%	54.38
KEEG mapper reconstruction	
KEEG orthology (KO)	2671
Metabolism protein	1487
Genetic information processing	690
Signaling and cellular processes	829
Carbohydrate metabolism	318
Amino acid metabolism	157
Nucleotide metabolism	104
Metabolism of cofactors and vitamins	134
Energy metabolism	101
EggNOG-Mapper	
COG	4349
Pfam	4186
GO	2540
CAZy	77
BIGG	1064

**Table 3:** Reference genomes of enterobacteria from GenBank, strain code, scientific name, and FastANI results.

	Genomes NCBI			FAST ANI	
Strain	Genbank	Scientific Name	ANI Score	Fragment Length	Total Fragment
Crenshaw	GCA_016027695.1	*E. asburiae*	87.8055	1250	1586
ATCC 35953	GCA_001521715.1	*E. asburiae*	87.8536	1241	1586
JM-458T.1	GCA_900180435.1	*E. asburiae*	87.8124	1282	1586
E1	GCA_008364625.1	*E. dykesii*	87.8021	1262	1586
DSM 16690	GCA_001729805.1	*E. roggenkampii*	87.6385	1258	1586
CCA6	GCA_009176645.1	*E. oligotrophicus*	87.547	1218	1586
WCHECL1597	GCA_002939185.1	*E. sichuanensis*	87.326	1248	1586
WCHECl-C4	GCA_001984825.2	*E. chengduensis*	87.3321	1295	1586
DSM-13645	GCA_001729765.1	*E. kobei*	87.1635	1222	1586
FDAARGOS 1428	GCA_019047785.1	*E. cancerogenus*	86.5672	1214	1586
EB-247	GCA_900324475.1	*E. bugandensis*	87.4329	1290	1586
ATCC BAA-2102	GCA_001654845.1	*E. soli*	86.5295	1222	1586
ATCC 23216	GCA_000735515.1	*Leclercia adecarboxylata*	83.6121	1043	1586
SB6411	GCA_902158555.1	*Klebsiella spallanzanii*	80.9556	840	1586
ATCC 8090	GCA_011064845.1	*Citrobacter freundii*	81.5917	820	1586

**Table 4:** Evaluation of hpc protein models from *Enterobacter* sp. UNJFSC003. Physicochemical analysis and signal peptide.

Modeling Server	Protein Modeling	Errat	Error/Warning/Plass	R. Plot%	Z-Score	SignalP	Num. aa	pI	Mol Weight	GRAVY
trRosseta	hpcB	97.87	2/4/3	95.4%	−11.36	No	283	5.72	31721.04	−0.228
trRosseta	hpcC	93.38	2/4/3	94.0%	−11.41	No	488	6.25	53130.86	−0.112
trRosseta	hpcD	83.49	1/3/4	94.6%	−4.79	No	126	5.82	14336.35	−0.202
trRosseta	hpcE	92.44	3/2/4	90.6%	−9.65	No	425	5.03	46227.37	−0.146
trRosseta	hpcG	93.44	0/4/4	91.8%	−6.87	No	267	5.69	29481.68	−0.081
trRosseta	hpcH	96.85	0/4/5	94.7%	−8.81	No	265	5.73	28175.33	0.107

## Data Availability

The sequence reads generated in this study have been submitted to the Sequence Read Archive (SRA) under the accession number SRR28705977. The assembled genome has been deposited to NCBI GenBank under the accession number JBAKHK000000000, the BioProject accession number PRJNA1076189, and the Biosample accession number SAMN39934077. The PROKKA annotation files have been published on Zenodo (DOI: https://doi.org/10.5281/zenodo.11069170).
